# Supplementary material for: Assessment of residential exposures to agricultural pesticides: A scoping review
Source: PLoS One. 2020 Apr 28;15(4):e0232258. doi: 10.1371/journal.pone.0232258 (PMC7188210; doi:10.1371/journal.pone.0232258)
Supplement: S1 Appendix — (DOCX) [file pone.0232258.s001.docx]

**References of articles selected in the scoping review**

Abdalla MH, Gutierrez-Mohamed ML, Farah IO. 2003. Association of pesticide exposure and risk of breast cancer mortality in Mississippi. Biomedical sciences instrumentation 39: 397–401.

Agopian AJ, Cai Y, Langlois PH, Canfield MA, Lupo PJ. 2013a. Maternal residential atrazine exposure and risk for choanal atresia and stenosis in offspring. Journal of Pediatrics 162:581–586; doi:10.1016/j.jpeds.2012.08.012.

Agopian AJ, Langlois PH, Cai Y, Canfield MA, Lupo PJ. 2013b. Maternal residential atrazine exposure and gastroschisis by maternal age. Maternal and Child Health Journal 17:1768–1775; doi:10.1007/s10995-012-1196-3.

Agopian AJ, Lupo PJ, Canfield MA, Langlois PH. 2013c. Case-Control Study of Maternal Residential Atrazine Exposure and Male Genital Malformations. American Journal of Medical Genetics, Part A 161:977–982; doi:10.1002/ajmg.a.35815.

Almberg KS, Turyk M, Jones RM, Anderson R, Graber J, Banda E, et al. 2014. A study of adverse birth outcomes and agricultural land use practices in Missouri. Environmental Research 134:420–426; doi:10.1016/j.envres.2014.06.016.

Aschengrau A, Ozonoff D, Coogan P, Vezina R, Heeren T, Zhang Y. 1996. Cancer risk and residential proximity to cranberry cultivation in Massachusetts. American journal of public health 86: 1289–96.

Babina K, Dollard M, Pilotto L, Edwards JW. 2012. Environmental exposure to organophosphorus and pyrethroid pesticides in South Australian preschool children: A cross sectional study. Environment International 48:109–120; doi:10.1016/j.envint.2012.07.007.

Bell EM, Hertz-Picciotto I, Beaumont JJ. 2001a. A case-control study of pesticides and fetal death due to congenital anomalies. Epidemiology 12:148–156; doi:10.1097/00001648-200103000-00005.

Bell EM, Hertz-Picciotto I, Beaumont JJ. 2001b. Case-cohort analysis of agricultural pesticide applications near maternal residence and selected causes of fetal death. American Journal of Epidemiology 154:702–710; doi:10.1093/aje/154.8.702.

Beranger R, Perol O, Bujan L, Faure E, Blain J, Le Cornet C, et al. 2014. Studying the impact of early life exposures to pesticides on the risk of testicular germ cell tumors during adulthood (TESTIS project): Study protocol. BMC CANCER 14; doi:10.1186/1471-2407-14-563.

Berenstein G, Nasello S, Beiguel É, Flores P, Di Schiena J, Basack S, et al. 2017. Human and soil exposure during mechanical chlorpyrifos, myclobutanil and copper oxychloride application in a peach orchard in Argentina. Science of the Total Environment 586:1254–1262; doi:10.1016/j.scitotenv.2017.02.129.

Bonvallot N, Tremblay-Franco M, Chevrier C, Canlet C, Warembourg C, Cravedi J-P, et al. 2013. Metabolomics Tools for Describing Complex Pesticide Exposure in Pregnant Women in Brittany (France). PLoS ONE 8; doi:10.1371/journal.pone.0064433.

Booth BJ, Ward MH, Turyk ME, Stayner LT. 2015. Agricultural crop density and risk of childhood cancer in the midwestern United States: An ecologic study. Environmental Health: A Global Access Science Source 14; doi:10.1186/s12940-015-0070-3.

Bradman A, Castorina R, Barr DB, Chevrier J, Harnly ME, Eisen EA, et al. 2011. Determinants of organophosphorus pesticide urinary metabolite levels in young children living in an agricultural community. International Journal of Environmental Research and Public Health 8:1061–1083; doi:10.3390/ijerph8041061.

Brender JD, Felkner M, Suarez L, Canfield MA, Henry JP. 2010. Maternal Pesticide Exposure and Neural Tube Defects in Mexican Americans. Annals of Epidemiology 20:16–22; doi:10.1016/j.annepidem.2009.09.011.

Brody JG, Aschengrau A, McKelvey W, Rudel RA, Swartz CH, Kennedy T. 2004. Breast cancer risk and historical exposure to pesticides from wide-area applications assessed with GIS. ENVIRONMENTAL HEALTH PERSPECTIVES 112:889–897; doi:10.1289/ehp.6845.

Brody JG, Vorhees DJ, Melly SJ, Swedis SR, Drivas PJ, Rudel RA. 2002. Using GIS and historical records to reconstruct residential exposure to large-scale pesticide application. Journal of Exposure Analysis and Environmental Epidemiology 12:64–80; doi:10.1038/sj/jea/7500205.

Brouwer M, Huss A, van der Mark M, Nijssen PCG, Mulleners WM, Sas AMG, et al. 2017. Environmental exposure to pesticides and the risk of Parkinson’s disease in the Netherlands. Environment International 107:100–110; doi:10.1016/j.envint.2017.07.001.

Brouwer M, Kromhout H, Vermeulen R, Duyzer J, Kramer H, Hazeu G, et al. 2018. Assessment of residential environmental exposure to pesticides from agricultural fields in the Netherlands. Journal of Exposure Science and Environmental Epidemiology 28:173–181; doi:10.1038/jes.2017.3.

Bruno Schiffers. 2018. Rapport des essais supervisés par Gembloux Agro-Bio Tech dans le cadre du projet PROPULPPP, “Objectivation de l’exposition des populations aux pulvérisations de produits phytopharmaceutiques en Wallonie.” 98.

Bukalasa JS, Brunekreef B, Brouwer M, Koppelman GH, Wijga AH, Huss A, et al. 2018. Associations of residential exposure to agricultural pesticides with asthma prevalence in adolescence: The PIAMA birth cohort. Environment International 121:435–442; doi:10.1016/j.envint.2018.09.029.

Bukalasa JS, Brunekreef B, Brouwer M, Vermeulen R, de Jongste JC, van Rossem L, et al. 2017. Proximity to agricultural fields as proxy for environmental exposure to pesticides among children: The PIAMA birth cohort. Science of the Total Environment 595:515–520; doi:10.1016/j.scitotenv.2017.03.269.

Butler Ellis MC, van den Berg F, Kennedy MC, O’Sullivan CM, Jacobs CM, Fragkoulis G, et al. 2016. The BROWSE model for predicting exposures of residents and bystanders to agricultural use of plant protection products: An overview. Biosystems Engineering 154:92–104; doi:10.1016/J.BIOSYSTEMSENG.2016.08.017.

Butler-Dawson J, Galvin K, Thorne PS, Rohlman DS. 2018. Organophosphorus pesticide residue levels in homes located near orchards. Journal of occupational and environmental hygiene 1–24; doi:10.1080/15459624.2018.1515489.

Butler-Ellis C. 2012. Bystander and resident exposures to pesticides used in agriculture: Recent work to update the model used to assess exposure of the public in the UK. Outlooks on Pest Management 23:7–12; doi:10.1564/23feb03.

Butler-Ellis MC, Kennedy MC, Kuster CJ, Alanis R, Tuck CR. 2018. Improvements in modelling bystander and resident exposure to pesticide spray drift: Investigations into new approaches for characterizing the ‘collection efficiency’’ of the human body.’ Annals of Work Exposures and Health 62:622–632; doi:10.1093/annweh/wxy017.

Butler-Ellis MC, Underwood B, Peirce MJ, Walker CT, Miller PCH, Butler Ellis MC, et al. 2010. Modelling the dispersion of volatilised pesticides in air after application for the assessment of resident and bystander exposure. Biosystems Engineering 107:149–154; doi:10.1016/j.biosystemseng.2010.08.002.

Butler-Ellis MC, van de Zande JC, van den Berg F, Kennedy MC, O’Sullivan CM, Jacobs CMCM, et al. 2016. The BROWSE model for predicting exposures of residents and bystanders to agricultural use of pesticides: Comparison with experimental data and other exposure models. Biosystems Engineering 154:92–104; doi:10.1016/j.biosystemseng.2016.08.017.

Carles C, Bouvier G, Esquirol Y, Piel C, Migault L, Pouchieu C, et al. 2017. Residential proximity to agricultural land and risk of brain tumor in the general population. Environmental Research 159:321–330; doi:10.1016/j.envres.2017.08.025.

Carmichael SL, Yang W, Ma C, Roberts E, Kegley S, English P, et al. 2016a. Joint effects of genetic variants and residential proximity to pesticide applications on hypospadias risk. Birth Defects Research Part A - Clinical and Molecular Teratology 106:653–658; doi:10.1002/bdra.23508.

Carmichael SL, Yang W, Roberts E, Kegley SE, Brown TJ, English PB, et al. 2016b. Residential agricultural pesticide exposures and risks of selected birth defects among offspring in the San Joaquin Valley of California. Birth Defects Research Part A - Clinical and Molecular Teratology 106:27–35; doi:10.1002/bdra.23459.

Carmichael SL, Yang W, Roberts E, Kegley SE, Padula AM, English PB, et al. 2014. Residential agricultural pesticide exposures and risk of selected congenital heart defects among offspring in the San Joaquin Valley of California. Environmental Research 135:133–138; doi:10.1016/j.envres.2014.08.030.

Carmichael SL, Yang W, Roberts EM, Kegley SE, Wolff C, Guo L, et al. 2013. Hypospadias and residential proximity to pesticide applications. Pediatrics 132:e1216–e1226; doi:10.1542/peds.2013-1429.

Carozza SE, Li B, Elgethun K, Whitworth R. 2008. Risk of childhood cancers associated with residence in agriculturally intense areas in the United States. Environmental Health Perspectives 116:559–565; doi:10.1289/ehp.9967.

Carozza SE, Li B, Wang Q, Horel S, Cooper S. 2009. Agricultural pesticides and risk of childhood cancers. International Journal of Hygiene and Environmental Health 212:186–195; doi:10.1016/j.ijheh.2008.06.002.

Castorina R, Bradman A, McKone TE, Barr DB, Harnly ME, Eskenazi B. 2003. Cumulative organophosphate pesticide exposure and risk assessment among pregnant women living in an agricultural community: A case study from the CHAMACOS cohort. Environmental Health Perspectives 111:1640–1648; doi:10.1289/ehp.5887.

Cha ES, Hwang S-S, Lee WJ. 2014. Childhood leukemia mortality and farming exposure in south korea: A national population-based birth cohort study. Cancer Epidemiology 38:401–407; doi:10.1016/j.canep.2014.05.003.

Chetty-Mhlanga S, Basera W, Fuhrimann S, Probst-Hensch N, Delport S, Mugari M, et al. 2018. A prospective cohort study of school-going children investigating reproductive and neurobehavioral health effects due to environmental pesticide exposure in the Western Cape, South Africa: Study protocol. BMC Public Health 18; doi:10.1186/s12889-018-5783-0.

Chevrier C, Serrano T, Lecerf R, Limon G, Petit C, Monfort C, et al. 2014. Environmental determinants of the urinary concentrations of herbicides during pregnancy: The PELAGIE mother-child cohort (France). Environment International 63:11–18; doi:10.1016/j.envint.2013.10.010.

Clary T, Ritz B. 2003. Pancreatic cancer mortality and organochlorine pesticide exposure in California, 1989-1996. American Journal of Industrial Medicine 43:306–313; doi:10.1002/ajim.10188.

Clementi M, Causin R, Marzocchi C, Mantovani A, Tenconi R. 2007. A study of the impact of agricultural pesticide use on the prevalence of birth defects in northeast Italy. Reproductive Toxicology 24:1–8; doi:10.1016/j.reprotox.2007.04.066.

Cockburn M, Mills P, Zhang X, Zadnick J, Goldberg D, Ritz B. 2011. Prostate cancer and ambient pesticide exposure in agriculturally intensive areas in California. American Journal of Epidemiology 173:1280–1288; doi:10.1093/aje/kwr003.

Coker E, Gunier R, Bradman A, Harley K, Kogut K, Molitor J, et al. 2017. Association between pesticide profiles used on agricultural fields near maternal residences during pregnancy and IQ at age 7 years. International Journal of Environmental Research and Public Health 14; doi:10.3390/ijerph14050506.

Cornelis C, Schoeters G, Kellen E, Buntinx F, Zeegers M. 2009. Development of a GIS-based indicator for environmental pesticide exposure and its application to a Belgian case-control study on bladder cancer. International Journal of Hygiene and Environmental Health 212:172–185; doi:10.1016/j.ijheh.2008.06.001.

Coronado GD, Holte S, Vigoren E, Griffith WC, Barr DB, Faustman E, et al. 2011. Organophosphate pesticide exposure and residential proximity to nearby fields: Evidence for the drift pathway. Journal of Occupational and Environmental Medicine 53:884–891; doi:10.1097/JOM.0b013e318222f03a.

Costanzini S, Teggi S, Bigi A, Ghermandi G, Filippini T, Malagoli C, et al. 2018. Atmospheric dispersion modelling and spatial analysis to evaluate population exposure to pesticides from farming processes. Atmosphere 9; doi:10.3390/atmos9020038.

Costello S, Cockburn M, Bronstein J, Zhang X, Ritz B. 2009. Parkinson’s disease and residential exposure to maneb and paraquat from agricultural applications in the central valley of California. American Journal of Epidemiology 169:919–926; doi:10.1093/aje/kwp006.

Cunha JP, Chueca P, Garcerá C, Moltó E. 2012. Risk assessment of pesticide spray drift from citrus applications with air-blast sprayers in Spain. Crop Protection 42:116–123; doi:10.1016/j.cropro.2012.06.001.

Dalvie MA, Sosan MB, Africa A, Cairncross E, London L. 2014. Environmental monitoring of pesticide residues from farms at a neighbouring primary and pre-school in the Western Cape in South Africa. Science of the Total Environment 466–467:1078–1084; doi:10.1016/j.scitotenv.2013.07.099.

Dereumeaux C, Saoudi A, Goria S, Wagner V, De Crouy-Chanel P, Pecheux M, et al. 2018. Urinary levels of pyrethroid pesticides and determinants in pregnant French women from the Elfe cohort. Environment International 119:89–99; doi:10.1016/j.envint.2018.04.042.

European Food Safety Authority (EFSA). 2014. Guidance on the assessment of exposure of operators, workers, residents and bystanders in risk assessment for plant protection products. EFSA Journal 12:3874; doi:10.2903/j.efsa.2014.3874.

Fenske RA, Lu C, Barr D, Needham L. 2002. Children’s exposure to chlorpyrifos and parathion in an agricultural community in central Washington State. Environmental Health Perspectives 110:549–553; doi:10.1289/ehp.02110549.

Ferri GM, Guastadisegno CM, Intranuovo G, Cavone D, Birtolo F, Cecinati V, et al. 2018. Maternal Exposure to Pesticides, Paternal Occupation in the Army/Police Force, and CYP2D6{*}4 Polymorphism in the Etiology of Childhood Acute Leukemia. Journal Of Pediatric Hematology Oncology 40:E207–E214; doi:10.1097/MPH.0000000000001105.

Galea KS, MacCalman L, Jones K, Cocker J, Teedon P, Cherrie JW, et al. 2015a. Comparison of residents’ pesticide exposure with predictions obtained using the UK regulatory exposure assessment approach. Regulatory Toxicology and Pharmacology 73:634–643; doi:10.1016/j.yrtph.2015.09.012.

Galea KS, MacCalman L, Jones K, Cocker J, Teedon P, Cherrie JW, et al. 2015b. Urinary biomarker concentrations of captan, chlormequat, chlorpyrifos and cypermethrin in UK adults and children living near agricultural land. Journal of Exposure Science and Environmental Epidemiology 25:623–631; doi:10.1038/jes.2015.54.

Garcia-Perez J, Morales-Piga A, Gomez J, Gomez-Barroso D, Tamayo-Uria I, Pardo Romaguera E, et al. 2016. Association between residential proximity to environmental pollution sources and childhood renal tumors. Environmental Research 147:405–414; doi:10.1016/j.envres.2016.02.036.

Gemmill A, Gunier RB, Bradman A, Eskenazi B, Harley KG. 2013. Residential proximity to methyl bromide use and birth outcomes in an agricultural population in California. Environmental Health Perspectives 121:737–743; doi:10.1289/ehp.1205682.

Gibbs JL, Yost MG, Negrete M, Fenske RA. 2017. Passive sampling for indoor and outdoor exposures to chlorpyrifos, azinphos-methyl, and oxygen analogs in a rural agricultural community. Environmental Health Perspectives 125:333–341; doi:10.1289/EHP425.

Glorennec P, Serrano T, Fravallo M, Warembourg C, Monfort C, Cordier S, et al. 2017. Determinants of children’s exposure to pyrethroid insecticides in western France. Environment International 104:76–82; doi:10.1016/j.envint.2017.04.007.

Gomez-Barroso D, Garcia-Perez J, Lopez-Abente G, Tamayo-Uria I, Morales-Piga A, Pardo Romaguera E, et al. 2016. Agricultural crop exposure and risk of childhood cancer: new findings from a case-control study in Spain. International Journal of Health Geographics 15; doi:10.1186/s12942-016-0047-7.

González-Alzaga B, Hernández AF, Rodríguez-Barranco M, Gómez I, Aguilar-Garduño C, López-Flores I, et al. 2015. Pre- and postnatal exposures to pesticides and neurodevelopmental effects in children living in agricultural communities from South-Eastern Spain. Environment International 85:229–237; doi:10.1016/j.envint.2015.09.019.

Gooijer YM, Hoftijser GW, Lageschaar LCC, Oerlemans A, Scheepers PTJ, Kivits CM, et al. 2019. Research on exposure of residents to pesticides in the Netherlands : OBO flower bulbs = Onderzoek Bestrijdingsmiddelen en Omwonenden. 381.

Gunier RB, Bradman A, Castorina R, Holland NT, Avery D, Harley KG, et al. 2017. Residential proximity to agricultural fumigant use and IQ, attention and hyperactivity in 7-year old children. Environmental Health Perspectives 125:358–365; doi:10.1016/j.envres.2017.06.036.

Gunier RB, Bradman A, Jerrett M, Smith DR, Harley KG, Austin C, et al. 2013. Determinants of manganese in prenatal dentin of shed teeth from CHAMACOS children living in an agricultural community. Environmental Science and Technology 47:11249–11257; doi:10.1021/es4018688.

Gunier RB, Jerrett M, Smith DR, Jursa T, Yousefi P, Camacho J, et al. 2014. Determinants of manganese levels in house dust samples from the CHAMACOS cohort. Science of the Total Environment 497–498:360–368; doi:10.1016/j.scitotenv.2014.08.005.

Gunier RB, Raanan R, Castorina R, Holland NT, Harley KG, Balmes JR, et al. 2018. Residential proximity to agricultural fumigant use and respiratory health in 7-year old children. ENVIRONMENTAL RESEARCH 164:93–99; doi:10.1016/j.envres.2018.02.022.

Gunier RB, Ward MH, Airola M, Bell EM, Colt J, Nishioka M, et al. 2011. Determinants of agricultural pesticide concentrations in carpet dust. Environmental Health Perspectives 119:970–976; doi:10.1289/ehp.1002532.

Harnly ME, Bradman A, Nishioka M, Mckone TE, Smith D, Mclaughlin R, et al. 2009. Pesticides in dust from homes in an agricultural area. Environmental Science and Technology 43:8767–8774; doi:10.1021/es9020958.

Hoffmann W, Terschüeren C, Heimpel H, Feller A, Butte W, Hostrup O, et al. 2008. Population-based research on occupational and environmental factors for leukemia and non Hodgkin’s lymphoma: The Northern Germany Leukemia and Lymphoma Study (NLL). American Journal of Industrial Medicine 51:246–257; doi:10.1002/ajim.20551.

Hogenkamp A, Vaal M, Heederik D. 2004. Pesticide exposure in dwellings near bulb growing fields in the Netherlands: An explorative study. Annals of Agriculture and Environmental Medicine 11: 149–153.

Hung C-C, Huang F-J, Yang Y-Q, Hsieh C-J, Tseng C-C, Yiin L-M. 2018. Pesticides in indoor and outdoor residential dust: a pilot study in a rural county of Taiwan. Environmental Science and Pollution Research 25:23349–23356; doi:10.1007/s11356-018-2413-4.

Institut Scientifique de Service Public. 2019. Objectivation de l’exposition des populations aux pulvérisations de produits phytopharmaceutiques en Wallonie et des mesures de protection destinées à limiter cette exposition - Etude PROPULPPP - Evaluation des risques pour la santé - Rapport n°04460/2018. 40.

Jones RR, Yu C-L, Nuckols JR, Cerhan JR, Airola M, Ross JA, et al. 2014. Farm residence and lymphohematopoietic cancers in the Iowa Women’s Health Study. Environmental Research 133:353–361; doi:10.1016/j.envres.2014.05.028.

Kennedy MC, Butler Ellis MC. 2017. Probabilistic modelling for bystander and resident exposure to pesticides using the Browse software. Biosystems Engineering 154:105–121; doi:10.1016/j.biosystemseng.2016.08.012.

Kennedy MC, Glass CR, Bokkers B, Hart ADM, Hamey PY, Kruisselbrink JW, et al. 2015. A European model and case studies for aggregate exposure assessment of pesticides. Food and Chemical Toxicology 79:32–44; doi:10.1016/j.fct.2014.09.009.

Koch D, Lu C, Fisker-Andersen J, Jolley L, Fenske RA. 2002. Temporal association of children’s pesticide exposure and agricultural spraying: Report of a longitudinal biological monitoring study. Environmental Health Perspectives 110:829–833; doi:10.1289/ehp.02110829.

Lammoglia S-K, Kennedy MC, Barriuso E, Alletto L, Justes E, Munier-Jolain N, et al. 2017. Assessing human health risks from pesticide use in conventional and innovative cropping systems with the BROWSE model. Environment International 105:66–78; doi:10.1016/j.envint.2017.04.012.

Langlois PH, Scheuerle A, Horel SA, Carozza SE. 2009. Urban versus rural residence and occurrence of septal heart defects in Texas. Birth Defects Research Part A - Clinical and Molecular Teratology 85:764–772; doi:10.1002/bdra.20586.

Larsen AE, Gaines SD, Deschênes O. 2017. Agricultural pesticide use and adverse birth outcomes in the San Joaquin Valley of California. Nature Communications 8:302; doi:10.1038/s41467-017-00349-2.

Lee P-C, Bordelon Y, Bronstein J, Ritz B. 2012. Traumatic brain injury, paraquat exposure, and their relationship to Parkinson disease. Neurology 79:2061–2066; doi:10.1212/WNL.0b013e3182749f28.

Lee P-C, Rhodes SL, Sinsheimer JS, Bronstein J, Ritz B. 2013. Functional paraoxonase 1 variants modify the risk of Parkinson’s disease due to organophosphate exposure. Environment International 56:42–47; doi:10.1016/j.envint.2013.03.004.

Levario-Carrillo M, Amato D, Ostrosky-Wegman P, González-Horta C, Corona Y, Sanin LH. 2004. Relation between pesticide exposure and intrauterine growth retardation. Chemosphere 55:1421–1427; doi:10.1016/j.chemosphere.2003.11.027.

Lin S, Marshall EG, Davidson GK. 1994. Potential parental exposure to pesticides and limb reduction defects. Scandinavian Journal of Work, Environment and Health 20:166–179; doi:10.5271/sjweh.1412.

Ling C, Liew Z, von Ehrenstein OS, Heck JE, Park AS, Cui X, et al. 2018. Prenatal Exposure to Ambient Pesticides and Preterm Birth and Term Low Birthweight in Agricultural Regions of California. Toxics 6; doi:10.3390/toxics6030041.

Lu CS, Fenske RA, Simcox NJ, Kalman D. 2000. Pesticide exposure of children in an agricultural community: Evidence of household proximity to farmland and take home exposure pathways. ENVIRONMENTAL RESEARCH 84:290–302; doi:10.1006/enrs.2000.4076.

Malagoli C, Costanzini S, Heck JE, Malavolti M, De Girolamo G, Oleari P, et al. 2016. Passive exposure to agricultural pesticides and risk of childhood leukemia in an Italian community. International Journal of Hygiene and Environmental Health 219:742–748; doi:10.1016/j.ijheh.2016.09.015.

Manthripragada AD, Costello S, Cockburn MG, Bronstein JM, Ritz B. 2010. Paraoxonase 1, agricultural organophosphate exposure, and Parkinson disease. Epidemiology 21:87–94; doi:10.1097/EDE.0b013e3181c15ec6.

Markel TA, Proctor C, Ying J, Winchester PD. 2015. Environmental pesticides increase the risk of developing hypertrophic pyloric stenosis. Journal of Pediatric Surgery 50:1283–1288; doi:10.1016/j.jpedsurg.2014.12.009.

Marusek JC, Cockburn MG, Mills PK, Ritz BR. 2006. Control selection and pesticide exposure assessment via GIS in prostate cancer studies. American Journal of Preventive Medicine 30:S109–S116; doi:10.1016/j.amepre.2005.09.002.

Meyer KJ, Reif JS, Rao Veeramachaneni DN, Luben TJ, Mosley BS, Nuckols JR. 2006. Agricultural pesticide use and hypospadias in Eastern Arkansas. Environmental Health Perspectives 114:1589–1595; doi:10.1289/ehp.9146.

Nigatu AW, Bratveit M, Moen BE. 2016. Self-reported acute pesticide intoxications in Ethiopia. BMC PUBLIC HEALTH 16; doi:10.1186/s12889-016-3196-5.

Nuckols JR, Gunier RB, Riggs P, Miller R, Reynolds P, Ward MH. 2007. Linkage of the California Pesticide Use Reporting Database with spatial land use data for exposure assessment. Environmental Health Perspectives 115:684–689; doi:10.1289/ehp.9518.

Ochoa-Acuna H, Carbajo C. 2009. Risk of limb birth defects and mother’s home proximity to cornfields. Science of the Total Environment 407:4447–4451; doi:10.1016/j.scitotenv.2009.04.028.

Parron T, Requena M, Hernandez AF, Alarcon R. 2011. Association between environmental exposure to pesticides and neurodegenerative diseases. Toxicology and Applied Pharmacology 256:379–385; doi:10.1016/j.taap.2011.05.006.

Paul KC, Chuang Y-H, Cockburn M, Bronstein JM, Horvath S, Ritz B. 2018a. Organophosphate pesticide exposure and differential genome-wide DNA methylation. Science of the Total Environment 645:1135–1143; doi:10.1016/j.scitotenv.2018.07.143.

Paul KC, Ling C, Lee A, To TM, Cockburn M, Haan M, et al. 2018b. Cognitive decline, mortality, and organophosphorus exposure in aging Mexican Americans. Environmental Research 160:132–139; doi:10.1016/j.envres.2017.09.017.

Petit C, Blangiardo M, Richardson S, Coquet F, Chevrier C, Cordier S. 2012. Association of environmental insecticide exposure and fetal growth with a bayesian model including multiple exposure sources: The PELAGIE mother-child cohort. American Journal of Epidemiology 175:1182–1190; doi:10.1093/aje/kwr422.

Petit C, Chevrier C, Durand G, Monfort C, Rouget F, Garlantezec R, et al. 2010. Impact on fetal growth of prenatal exposure to pesticides due to agricultural activities: A prospective cohort study in Brittany, France. Environmental Health: A Global Access Science Source 9; doi:10.1186/1476-069X-9-71.

Pivato A, Barausse A, Zecchinato F, Palmeri L, Raga R, Lavagnolo MC, et al. 2015. An integrated model-based approach to the risk assessment of pesticide drift from vineyards. Atmospheric Environment 111:136–150; doi:10.1016/j.atmosenv.2015.04.005.

Plascak JJ, Griffith WC, Workman T, Smith MN, Vigoren E, Faustman EM, et al. 2018. Evaluation of the relationship between residential orchard density and dimethyl organophosphate pesticide residues in house dust. Journal of exposure science & environmental epidemiology; doi:10.1038/s41370-018-0074-5.

Povedano M, Saez M, Martínez-Matos J-A, Barceló MA. 2018. Spatial Assessment of the Association between Long-Term Exposure to Environmental Factors and the Occurrence of Amyotrophic Lateral Sclerosis in Catalonia, Spain: A Population-Based Nested Case-Control Study. Neuroepidemiology 51:33–49; doi:10.1159/000489664.

Raanan R, Gunier RB, Balmes JR, Beltran AJ, Harley KG, Bradman A, et al. 2017. Elemental sulfur use and associations with pediatric lung function and respiratory symptoms in an agricultural community (California, USA). Environmental Health Perspectives 125; doi:10.1289/EHP528.

Ramis R, Tamayo-Uria I, Gómez-Barroso D, López-Abente G, Morales-Piga A, Pardo Romaguera E, et al. 2017. Risk factors for central nervous system tumors in children: New findings from a casecontrol study. PLoS ONE 12; doi:10.1371/journal.pone.0171881.

Rappazzo KM, Warren JL, Meyer RE, Herring AH, Sanders AP, Brownstein NC, et al. 2016. Maternal residential exposure to agricultural pesticides and birth defects in a 2003 to 2005 North Carolina birth cohort. Birth Defects Research Part A - Clinical and Molecular Teratology 106:240–249; doi:10.1002/bdra.23479.

Razi S, Rezaeian M, Dehkordi FG, Manshoori A, Goujani R, Vazirinejad R. 2016. Exposure to pistachio pesticides and stillbirth: a case-control study. Epidemiology and health 38:e2016016; doi:10.4178/epih.e2016016.

Reynolds P, Hurley SE, Goldberg DE, Yerabati S, Gunier RB, Hertz A, et al. 2004. Residential proximity to agricultural pesticide use and incidence of breast cancer in the California Teachers Study cohort. Environmental Research 96:206–218; doi:10.1016/j.envres.2004.03.001.

Reynolds P, Hurley SE, Gunier RB, Yerabati S, Quach T, Hertz A. 2005a. Residential proximity to agricultural pesticide use and incidence of breast cancer in California, 1988-1997. Environmental health perspectives 113:993–1000; doi:10.1289/ehp.7765.

Reynolds P, Von Behren J, Gunier R, Goldberg DE, Hertz A. 2005b. Agricultural pesticides and lymphoproliferative childhood cancer in California. Scandinavian Journal of Work, Environment and Health 31: 46–54.

Reynolds P, Von Behren J, Gunier RB, Goldberg DE, Harnly M, Hertz A. 2005c. Agricultural pesticide use and childhood cancer in California. Epidemiology 16:93–100; doi:10.1097/01.ede.0000147119.32704.5c.

Reynolds P, Von Behren J, Gunier RB, Goldberg DE, Hertz A, Harnly ME. 2002. Childhood cancer and agricultural pesticide use: an ecologic study in California. Environmental health perspectives 110:319–24; doi:10.1289/ehp.02110319.

Ritz B, Costello S. 2006. Geographic model and biomarker-derived measures of pesticide exposure and Parkinson’s disease. F. Mehlman, MA and Soffritti, M and Landrigan, P and Bingham, E and Belpoggi, ed Living in a chemical world: framing the future in light of the past 1076:378–387; doi:10.1196/annals.1371.074.

Ritz B, Yu F. 2000. Parkinson’s disease mortality and pesticide exposure in California 1984-1994. International Journal of Epidemiology 29:323–329; doi:10.1093/ije/29.2.323.

Ritz BR, Manthripragada AD, Costello S, Lincoln SJ, Farrer MJ, Cockburn M, et al. 2009. Dopamine transporter genetic variants and pesticides in Parkinson’s disease. Environmental Health Perspectives 117:964–969; doi:10.1289/ehp.0800277.

Roberts EM, English PB, Grether JK, Windham GC, Somberg L, Wolff C. 2007. Maternal residence near agricultural pesticide applications and autism spectrum disorders among children in the California Central Valley. Environmental Health Perspectives 115:1482–1489; doi:10.1289/ehp.10168.

Rowe C, Gunier R, Bradman A, Harley KG, Kogut K, Parra K, et al. 2016. Residential proximity to organophosphate and carbamate pesticide use during pregnancy, poverty during childhood, and cognitive functioning in 10-year-old children. Environmental Research 150:128–137; doi:10.1016/j.envres.2016.05.048.

Royster MO, Hilborn ED, Barr D, Carty CL, Rhoney S, Walsh D. 2002. A pilot study of global positioning system/geographical information system measurement of residential proximity to agricultural fields and urinary organophosphate metabolite concentrations in toddlers. journal of exposure analysis and environmental epidemiology 12:433–440; doi:10.1038/sj.jea.7500247.

Rull RP, Gunier R, Von Behren J, Hertz A, Crouse V, Buffler PA, et al. 2009. Residential proximity to agricultural pesticide applications and childhood acute lymphoblastic leukemia. Environmental Research 109:891–899; doi:10.1016/j.envres.2009.07.014.

Rull RP, Ritz B, Shaw GM. 2006. Neural tube defects and maternal residential proximity to agricultural pesticide applications. American Journal of Epidemiology 163:743–753; doi:10.1093/aje/kwj101.

Ryberg MW, Rosenbaum RK, Mosqueron L, Fantke P. 2018. Addressing bystander exposure to agricultural pesticides in life cycle impact assessment. Chemosphere 197:541–549; doi:10.1016/j.chemosphere.2018.01.088.

Sade MY, Zlotnik Y, Kloog I, Novack V, Peretz C, Ifergane G. 2015. Parkinson’s Disease Prevalence and Proximity to Agricultural Cultivated Fields. Parkinsons Disease; doi:10.1155/2015/576564.

Saez M, Barceló MA, Farrerons M, López-Casasnovas G. 2018. The association between exposure to environmental factors and the occurrence of attention-deficit/hyperactivity disorder (ADHD). A population-based retrospective cohort study. Environmental Research 166:205–214; doi:10.1016/j.envres.2018.05.009.

Sagiv SK, Harris MH, Gunier RB, Kogut KR, Harley KG, Deardorff J, et al. 2018. Prenatal organophosphate pesticide exposure and traits related to autism spectrum disorders in a population living in proximity to agriculture. Environmental Health Perspectives 126; doi:10.1289/EHP2580.

Schreinemachers DM. 2003. Birth malformations and other adverse perinatal outcomes in four U.S. Wheat-producing states. Environmental health perspectives 111:1259–64; doi:10.1289/ehp.5830.

Schwartz DA, LoGerfo JP. 1988. Congenital limb reduction defects in the agricultural setting. American Journal of Public Health 78: 654–659.

Shaw GM, Yang W, Roberts E, Kegley SE, Padula A, English PB, et al. 2014. Early pregnancy agricultural pesticide exposures and risk of gastroschisis among offspring in the San Joaquin Valley of California. Birth Defects Research Part A - Clinical and Molecular Teratology 100:686–694; doi:10.1002/bdra.23263.

Shaw GM, Yang W, Roberts EM, Aghaeepour N, Mayo JA, Weber KA, et al. 2018a. Residential agricultural pesticide exposures and risks of preeclampsia. Environmental Research 164:546–555; doi:10.1016/j.envres.2018.03.020.

Shaw GM, Yang W, Roberts EM, Kegley SE, Stevenson DK, Carmichael SL, et al. 2018b. Residential Agricultural Pesticide Exposures and Risks of Spontaneous Preterm Birth. Epidemiology 29:8–21; doi:10.1097/EDE.0000000000000757.

Shelton JF, Geraghty EM, Tancredi DJ, Delwiche LD, Schmidt RJ, Ritz B, et al. 2014. Neurodevelopmental disorders and prenatal residential proximity to agricultural pesticides: The charge study. Environmental Health Perspectives 122:1103–1109; doi:10.1289/ehp.1307044.

Silva MH, Dong MH. 2015. The Health Risk Assessment Performed in California for the Herbicide Simazine: A Case Study. Human and Ecological Risk Assessment 21:1496–1517; doi:10.1080/10807039.2014.958022.

Suarez-Lopez JR, Butcher CR, Gahagan S, Checkoway H, Alexander BH, Al-Delaimy WK. 2018a. Acetylcholinesterase activity and time after a peak pesticide-use period among Ecuadorian children. International Archives of Occupational and Environmental Health 91:175–184; doi:10.1007/s00420-017-1265-4.

Suarez-Lopez JR, Hong V, McDonald KN, Suarez-Torres J, López D, De La Cruz F. 2018b. Home proximity to flower plantations and higher systolic blood pressure among children. International Journal of Hygiene and Environmental Health 221:1077–1084; doi:10.1016/j.ijheh.2018.08.006.

Takser L, Lafond J, Bouchard M, St-Amour G, Mergler D. 2004. Manganese levels during pregnancy and at birth: Relation to environmental factors and smoking in a Southwest Quebec population. Environmental Research 95:119–125; doi:10.1016/j.envres.2003.11.002.

Thompson JA, Carozza SE, Zhu L. 2008. Geographic risk modeling of childhood cancer relative to county-level crops, hazardous air pollutants and population density characteristics in Texas. ENVIRONMENTAL HEALTH 7; doi:10.1186/1476-069X-7-45.

Uysal M, Bozcuk H, Karakilinc H, Goksu S, Tatli AM, Gunduz S, et al. 2013. Pesticides and cancer: The first incidence study conducted in Turkey. Journal of Environmental Pathology, Toxicology and Oncology 32:245–249; doi:10.1615/JEnvironPatholToxicolOncol.2013008232.

Van den Berg F, Jacobs CMJ, Butler-Ellis MC, Spanoghe P, Doan Ngoc K, Fragkoulis G. 2016. Modelling exposure of workers, residents and bystanders to vapour of plant protection products after application to crops. Science of the Total Environment 573:1010–1020; doi:10.1016/j.scitotenv.2016.08.180.

Vargas RJ, Guzmán-Quilo C, Del Valle JM, Saldaña DG. 2014. Pesticide exposure and risk of acute lymphoblastic leukemia in children from Quetzaltenango, Guatemala. Pharmacologyonline 1: 16–20.

Vinceti M, Filippini T, Violi F, Rothman KJ, Costanzini S, Malagoli C, et al. 2017. Pesticide exposure assessed through agricultural crop proximity and risk of amyotrophic lateral sclerosis. Environmental Health: A Global Access Science Source 16; doi:10.1186/s12940-017-0297-2.

VoPham T, Brooks MM, Yuan J-M, Talbott EO, Ruddell D, Hart JE, et al. 2015. Pesticide exposure and hepatocellular carcinoma risk: A case-control study using a geographic information system (GIS) to link SEER-Medicare and California pesticide data. Environmental Research 143:68–82; doi:10.1016/j.envres.2015.09.027.

Walker KM, Carozza S, Cooper S, Elgethun K. 2007. Childhood cancer in Texas counties with moderate to intense agricultural activity. Journal of agricultural safety and health 13: 9–24.

Wan N, Lin G. 2016. Parkinson’s Disease and Pesticides Exposure: New Findings From a Comprehensive Study in Nebraska, USA. Journal of Rural Health 32:303–313; doi:10.1111/jrh.12154.

Wang A, Cockburn M, Ly TT, Bronstein JM, Ritz B. 2014. The association between ambient exposure to organophosphates and Parkinson’s disease risk. Occupational and Environmental Medicine 71:275–281; doi:10.1136/oemed-2013-101394.

Wang A, Costello S, Cockburn M, Zhang X, Bronstein J, Ritz B. 2011. Parkinson’s disease risk from ambient exposure to pesticides. European Journal of Epidemiology 26:547–555; doi:10.1007/s10654-011-9574-5.

Wang W, Huang M-J, Wu F-Y, Kang Y, Wang H-S, Cheung KC, et al. 2013. Risk assessment of bioaccessible organochlorine pesticides exposure via indoor and outdoor dust. Atmospheric Environment 77:525–533; doi:10.1016/j.atmosenv.2013.04.071.

Ward MH, Lubin J, Giglierano J, Colt JS, Wolter C, Bekiroglu N, et al. 2006. Proximity to crops and residential to agricultural herbicides in Iowa. Environmental Health Perspectives 114:893–897; doi:10.1289/ehp.8770.

Ward MH, Nuckols JR, Weigel SJ, Maxwell SK, Cantor KP, Miller RS. 2000. Identifying populations potentially exposed to agricultural pesticides using remote sensing and a geographic information system. Environmental Health Perspectives 108: 5–12.

Wesseling C, Antich D, Hogstedt C, Rodríguez AC, Ahlbom A. 1999. Geographical differences of cancer incidence in Costa Rica in relation to environmental and occupational pesticide exposure. International Journal of Epidemiology 28:365–374; doi:10.1093/ije/28.3.365.

Winchester P, Proctor C, Ying J. 2016. County-level pesticide use and risk of shortened gestation and preterm birth. Acta Paediatrica, International Journal of Paediatrics 105:e107–e115; doi:10.1111/apa.13288.

Wong HL, Garthwaite DG, Ramwell CT, Brown CD. 2017. How does exposure to pesticides vary in space and time for residents living near to treated orchards? Environmental Science and Pollution Research 24:26444–26461; doi:10.1007/s11356-017-0064-5.

Xiang HY, Nuckols JR, Stallones L. 2000. A geographic information assessment of birth weight and crop production patterns around mother’s residence. Environmental Research 82:160–167; doi:10.1006/enrs.1999.4009.

Yang W, Carmichael SL, Roberts EM, Kegley SE, Padula AM, English PB, et al. 2014. Residential agricultural pesticide exposures and risk of neural tube defects and orofacial clefts among offspring in the San Joaquin Valley of California. American Journal of Epidemiology 179:740–748; doi:10.1093/aje/kwt324.

Yesavage JA, Sheikh J, Noda A, Murphy G, O’Hara R, Hierholzer R, et al. 2006. Spatial Test for Agricultural Pesticide “Blow-In” Effect on Prevalence of Parkinson’s Disease. Journal of Geriatric Psychiatry and Neurology 19:32–35; doi:10.1177/0891988705284707.
