# Supplementary material for: Assessment of residential exposures to agricultural pesticides: A scoping review
Source: PLoS One. 2020 Apr 28;15(4):e0232258. doi: 10.1371/journal.pone.0232258 (PMC7188210; doi:10.1371/journal.pone.0232258)
Supplement: S2 Appendix — (DOCX) [file pone.0232258.s002.docx]

**Appendix 2 – Table of health outcomes investigated in the epidemiological studies in the articles selected for scoping review (N=98)**

| **Health outcomes** | **n (N=98)** | **%** |
| --- | --- | --- |
| Blood cancer | 14 | 14.3 |
| Parkinson's disease | 10 | 10.2 |
| All cancer (types not specified) | 5 | 5.1 |
| Liver cancer | 5 | 5.1 |
| Brain cancer | 5 | 5.1 |
| Breast cancer | 5 | 5.1 |
| Central nervous system (CNS) and miscellaneous intracranial and intraspinal neoplasms | 5 | 5.1 |
| Kidney cancer | 5 | 5.1 |
| Preterm births | 5 | 5.1 |
| Limb reduction defects | 4 | 4.1 |
| Hypospadias | 4 | 4.1 |
| Neural tube defect (anencephaly/iniencephaly) | 4 | 4.1 |
| Neuroblastoma and other peripheral nervous cell tumors | 4 | 4.1 |
| Gene polymorphism | 4 | 4.1 |
| Congenital heart defects | 4 | 4.1 |
| Fetal growth indicators (birth lenght, head circumference, …) | 4 | 4.1 |
| Gastrointestinal anomalies | 3 | 3.1 |
| Low birth weight | 3 | 3.1 |
| Cognitive performance | 3 | 3.1 |
| Bone cancer | 3 | 3.1 |
| Autism spectrum disorders | 3 | 3.1 |
| Craniofacial congenital malformations | 3 | 3.1 |
| Spina bifida | 3 | 3.1 |
| Full-Scale Intelligence Quotient (FSIQ) | 3 | 3.1 |
| Sarcomas | 3 | 3.1 |
| Retinoblastoma | 2 | 2.0 |
| Colorectal cancer | 2 | 2.0 |
| Pancreas cancer | 2 | 2.0 |
| Small penis | 2 | 2.0 |
| Bladder cancer | 2 | 2.0 |
| Biological effect | 2 | 2.0 |
| Carcinomas and melanomas | 2 | 2.0 |
| Other respiratory symptoms | 2 | 2.0 |
| Urogenital congenital malformations | 2 | 2.0 |
| Amyotrophic lateral sclerosis | 2 | 2.0 |
| All types of malformations | 2 | 2.0 |
| Fetal death | 2 | 2.0 |
| Prostate cancer | 1 | 1.0 |
| Attention-deficit/hyperactivity disorder (ADHD) | 1 | 1.0 |
| Asthma | 1 | 1.0 |
| Anotia/microtia | 1 | 1.0 |
| Birth outcomes (all included) | 1 | 1.0 |
| Anorectal atresia/stenosis | 1 | 1.0 |
| Anomalies of the urinary system | 1 | 1.0 |
| Anomalies of the lung | 1 | 1.0 |
| Allergy | 1 | 1.0 |
| Respiratory congenital malformations | 1 | 1.0 |
| Testicular germ cell tumors (TGCT) | 1 | 1.0 |
| Gastroschisis | 1 | 1.0 |
| Malignant melanoma | 1 | 1.0 |
| Mortality | 1 | 1.0 |
| Multiple congenital anomalies | 1 | 1.0 |
| Musculoskeletal defects | 1 | 1.0 |
| Intrauterine growth retardation | 1 | 1.0 |
| Neonatal death under 24 hours | 1 | 1.0 |
| Hypertrophic pyloric stenosis | 1 | 1.0 |
| Choanal Atresia or stenosis | 1 | 1.0 |
| Neurological disorders (all included) | 1 | 1.0 |
| Reproductive health outcomes (testicular size, breast size) | 1 | 1.0 |
| Acute pesticide intoxications | 1 | 1.0 |
| Encephalocele | 1 | 1.0 |
| Diaphragmatic hernia | 1 | 1.0 |
| Cryptorchidism | 1 | 1.0 |
| Craniosynostosis | 1 | 1.0 |
| Preeclampsia | 1 | 1.0 |
| Lung cancer | 1 | 1.0 |
| Cognitive Decline | 1 | 1.0 |
| Genome-wide methylation | 1 | 1.0 |
