# Supplementary material for: Assessment of residential exposures to agricultural pesticides: A scoping review
Source: PLoS One. 2020 Apr 28;15(4):e0232258. doi: 10.1371/journal.pone.0232258 (PMC7188210; doi:10.1371/journal.pone.0232258)
Supplement: Attachment — Submitted filename: Clarify resident exposure.docx [file pone.0232258.s004.docx]

Great article, well written, I only have some minor suggestions to consider for discussion, to enrich the paper. See my remarks here:

Clarify resident exposure

Clarify why children of farmers are more exposed. The level of farmer children is twice time more? Ten times more? What is the variability/distribution in normal resident exposure and farmers residents exposure.

Residential exposure in EU might be totally different compared to residential exposure in US. Spray technology is different, treated farm area is different, type of used pesticides are different. There is a also a change in pesticides authorized for use in 2000 and 2019.

Chronic health effects from pesticides use may also date from pesticide use before 2000.

Young residents (body height) in 2000 are not equal to young residents in 2019, a different population is exposed in a different way over the years.

Some of the selected studies may be risk based studies and have a bias. The study motivation may come from an observation that certain health effects occur in a certain region. If the set-up is neutral then it may cover problematic and non-problematic areas. The size of the survey, local, regional national is important for not being biased.

*A wide range of active substances were included, with a quasi-equal representation of herbicides (n=35, 35.7%), insecticides (n=32, 32.7%) and fungicides (n=30, 30.6%). The number of substances included could be very different from one study to another, with a mean of 179 over a 190 range of 1 to 850 pesticides.*

This looks quite crucial if you want to do statements about pesticides. Which pesticides were used in which area and what is known about their toxicity (chronic effects). A different authorization in different regions in the world is a determining factor.

*Distances or buffer radiuses considered ranged from 0 to 8,000 meters. The smallest metric around the residence considered in the analysis (resolution) was generally less than 500 m.*

This all depends upon the local situation. I am not aware of a definition talking about resident exposure on a distance of 8 km. This is exposure of people living in a country side versus city/forest. Talking about that distance in Europe is rather unrealistic to consider, as another farmer may live and work more close.

*Developing the use of dispersion models could bring improvements to the assessment of residential exposure.*

I think there is confusion between acute and chronic effects. A dispersion model is a simplification of the reality. If you talk about this modelling you have to integrate a long term situation, in order to explain the chronic diseases of this article. As far as I understand the existing models, they only describe the situation of one application. This results in an acute exposure / symptoms.

*because a possible cocktail effect may overestimate health effects*

Above cocktail effects a quantification of exposure due to pesticide residues in food compared to the level of residents exposure would benefit heavily this article. What is the expected variability in exposure and are pesticides in food overruling the residential exposure. Is residential exposure higher compared to food exposure? What is the evidence for that.
