# Supplementary material for: Assessment of residential exposures to agricultural pesticides: A scoping review
Source: PLoS One. 2020 Apr 28;15(4):e0232258. doi: 10.1371/journal.pone.0232258 (PMC7188210; doi:10.1371/journal.pone.0232258)
Supplement: Attachment — Submitted filename: Response to reviewers.docx [file pone.0232258.s005.docx]

**Response to reviewers**

**Reference of the manuscript: PONE-D-20-00094 - Assessment of residential exposures to agricultural pesticides: a scoping review**

We firstly thank the academic editor and the reviewer for their valuable comments and suggestions. We have made changes to the article in an effort to make it more intelligible. The modifications are described hereafter, and we give a point-by-point response to the reviewers’ concerns.

**A/ Response to the Academic editor**

1. **Clarify resident exposure: Clarify why children of farmers are more exposed. The level of farmer children is twice time more? Ten times more? What is the variability/distribution in normal resident exposure and farmers residents exposure.**

Children of farmers are more exposed than children of non-agricultural families because of the take-home pathways. We referred to paraoccupational exposures in the line 132-135 but not in the discussion. In order to clarify this point, we made the following change (line 365-366 in the initial manuscript):

“*Only the number of farmworkers living in the house was well identified as a major contributor to pesticide exposure, mainly because of the take-home pesticide exposure pathway, as already demonstrated in previous reviews*”. We have not expanded the conclusions on the research in this field as our main objective was only to map the existing literature. We referred however to two systematic reviews dealing with the take-home exposure (*Hyland et al., 2018*) and (*Deziel et al., 2015*).

2. **Residential exposure in EU might be totally different compared to residential exposure in US. Spray technology is different, treated farm area is different, type of used are different. There is a also a change in pesticides authorized for use in 2000 and 2019.**

We agree and we added a sentence about these differences (line 354 of the original manuscript):

*“Variations exist between countries in spraying technologies, treated areas, types of pesticides authorized and used. These parameters may have also changed over time within a given country”.*

3. **Chronic health effects from pesticides use may also date from pesticide use before 2000.**

Indeed, it is crucial to take into account changes over time in exposure to study the health effects of pesticides. Otherwise, this could lead to errors in the risk assessment. As the main objective of this scoping review was to map the existing literature, we did not analyze the biases faced by the epidemiological studies and cannot conclude in the reliability of the methods used to study chronic health effects. However, we can see in Table 2 that the average duration of exposure considered in the selected studies was 7.5 years and the maximum of 50 years.

4. **Young residents (body height) in 2000 are not equal to young residents in 2019, a different population is exposed in a different way over the years.**

We agree with this comment. We consequently added the following sentence in the section “Health effects linked to pesticide exposure” (line 438 in the original document):

“*The characteristics of the populations living near agricultural areas can also vary greatly depending on the period and the geographical area under consideration*.”

5. **Some of the selected studies may be risk based studies and have a bias. The study motivation may come from an observation that certain health effects occur in a certain region. If the set-up is neutral then it may cover problematic and non-problematic areas. The size of the survey, local, regional national is important for not being biased.**

We chose to *a priori* exclude the cluster studies in order to avoid this situation. To clarify this point we added a sentence in the section “Methodology” (line 136 in the original manuscript): “*Finally, we excluded epidemiological publications focused on clusters of cases*”.

6. ***“A wide range of active substances were included, with a quasi-equal representation of herbicides (n=35, 35.7%), insecticides (n=32, 32.7%) and fungicides (n=30, 30.6%). The number of substances included could be very different from one study to another, with a mean of 179 over a 190 range of 1 to 850 pesticides*.” This looks quite crucial if you want to do statements about pesticides. Which pesticides were used in which area and what is known about their toxicity (chronic effects). A different authorization in different regions in the world is a determining factor.**

This remark joins that made in point #2. Corrections have been made in order to highlight the variability in the use of pesticides in space and time (cf. new paragraph added line 354 of the original manuscript).

7. ***“Distances or buffer radiuses considered ranged from 0 to 8,000 meters. The smallest metric around the residence considered in the analysis (resolution) was generally less than 500 m.”* This all depends upon the local situation. I am not aware of a definition talking about resident exposure on a distance of 8 km. This is exposure of people living in a country side versus city/forest. Talking about that distance in Europe is rather unrealistic to consider, as another farmer may live and work more close.**

The distances or buffer radiuses - ranging from 0 to 8,000 meters - were those defined by the authors to study the impact of the pesticides’ emission on health effects. The “8,000 meters” refers to a study conducted by Gunier et al. (2018) entitled “*Residential proximity to agricultural fumigant use and respiratory health in 7-year old children*”. The authors estimated the use of fumigant near the home by Geographic Information Systems (GIS) using the PUR dataset, for combination of distance from the residence (buffer radii of 3, 5, and 8 km). They found no significant associations between residential proximity to use of fumigants and respiratory symptoms or use of asthma medication. But other studies have conducted large-scale assessments of the pesticide exposure. As specified in line 220 of the original document, 4.3% found a positive association for a distance greater than 1,000 m. As developed in the Discussion, scoping reviews are not made to assess the quality of the studies selected (we did not perform any analysis of the studies’ biases) and it is not possible to interpret the meaning of the results we present any further.

8. **“*Developing the use of dispersion models could bring improvements to the assessment of residential exposure*.” I think there is confusion between acute and chronic effects. A dispersion model is a simplification of the reality. If you talk about this modelling you have to integrate a long term situation, in order to explain the chronic diseases of this article. As far as I understand the existing models, they only describe the situation of one application. This results in an acute exposure / symptoms.**

We agree that this could be an important limitation in the use of existing modelling tools. However, taking into account several treatments in a landscape is under development in recent studies. The limitation then is to get all the input data required and to be able to run simulations on a rather large scale and period of time. To specify this limitation, we changed the original sentence lines 403-405 of the initial manuscript as follows:

“*However, some of these models demand a lot of input data (meteorological and topographical data, amounts of pesticides applied, etc.) and their computing power can limit their use in ~~large~~ epidemiological studies involving many subjects or assessing long-term exposure.*”

9. **“*Because a possible cocktail effect may overestimate health effects*”. Above cocktail effects a quantification of exposure due to pesticide residues in food compared to the level of residents exposure would benefit heavily this article. What is the expected variability in exposure and are pesticides in food overruling the residential exposure. Is residential exposure higher compared to food exposure? What is the evidence for that.**

We totally agree with the importance to assess the relative contribution of the different exposure pathways in non-agricultural populations (drift, diet, take-home, and residential pathways), especially in order to develop effective and proportionate preventive interventions. Unfortunately, our scoping review does not enable us to answer this question. This should be investigated in future studies specifically designed to take into account all the potential biases linked to this kind of evaluation.

**B/ Response to the Reviewer #1**

1. **Line 46: is there a more international publication (e.g. sth from OECD of WHO) instead of (2)? Is a citation actually needed?**

**line 50: ref (3) does not seem to be the most dedicated paper pathways.**

We agree that the citation of an international publication in English would be more appropriate, we thus replaced the reference (2) and (3) by a reference of a European report using the following citation: *FOCUS (2008). “Pesticides in Air: Considerations for Exposure Assessment”. Report of the FOCUS Working Group on Pesticides in Air, EC Document Reference SANCO/10553/2006 Rev 2 June 2008. 327 pp.*

2. **Lines 53-56: How do you conclude from only paper (4) what is said about farmworkers vs residents?**

We chose to cite Kim et al. (2018), because this review is a comprehensive literature review including relatively recent publications (from 2010 to 2017). Among the 87 studies included in this review, most were conducted in occupational settings. Fewer publications focused on non-agricultural populations. However, no statistics on this precise point were given by the authors of this review. No comparison of health effects was made between farmworkers and residents.

3. **Line 58-59: who concluded there is no consensus?**

Indeed, the term "consensus" does not seem appropriate. We proposed the following amendment: *“Currently, there ~~is no consensus in the scientific literature on the definition of ‘residents’~~ no harmonized definition of the term “resident” in the scientific literature.*”

4. **Line 77: OPEX guidance does not say "only if the risk to humans can be considered as negligible" in these words. Also, would ref (1) not be a more precise source for this?**

We agree with this remark. We changed the reference (5) at the line 77 of the manuscript and modified the original sentence in order to respect the exact terms used in the European directive:

“*A substance will be authorized on the market only if the risk to humans can be considered as negligible” became “Substances will be authorized on the market only if they are not expected to have any harmful effect on human ~~the risk to humans~~*”.

5. **Lines 77-79: Are two messages mixed here? "including monitoring of the environment": is monitoring a preventive measure? Or, is understanding of residents' pathways and levels of exposure essential for monitoring of the environment (for the sake of the environment)??**

We agree that this formulation is confusing. We were referring to routine environmental monitoring of pesticides in the air. We rephrased the sentence (line 76 of the initial manuscript) as follows:

*“Finally, exposure assessment is needed in order to propose preventive operational measures to protect populations, including routine monitoring of ~~the environment~~ pesticides in the air.”*

6. **Line 335-336: title does not seem to cover the sections' messages. Reword?**

We changed the initial sections' messages “*Influence of agricultural sprayings on residential pesticide exposure*” into “*Evidence of Agricultural drift pathway*”.

7. **Lines 418 - 449: is this section needed? Health effects as such are not the focus. The part devoted to Gunier et al (2017) is not very clear to me. It has to do with identifiying risk mitigation measures, but I am not sure why this study is highlighted primarily on its conclusions. I think there is much more information on risk reduction measures (distance, barriers) out there.**

One of the main findings of this review is that the exposure assessment was conducted primarily for epidemiological studies to investigate possible links between pesticide exposure and adverse health effects. Therefore, we found useful to have a short comment on this

However, in order to clarify, we have shortened and reworded the section referring to Gunier et al (2017) with the following modifications: *“In the same way, Gunier et al. (2017) had previously concluded ~~it was a complex matter to identify a correct buffer around schools in agricultural areas that could be sufficiently protective against potential adverse effects. Indeed, given the very large number of substances that can be applied to crops, it is not possible to determine a single distance that could be protective for all of them. In addition, the more sensitive endpoint of children’s health could have not yet been studied in the scientific literature. Finally, perimeters could differ for pesticide mixtures because a possible cocktail effect may overestimate health effects.~~  that the determination of the relevant safety distance or buffer around schools in agricultural areas should be based on the published literature on pesticide exposure and adverse health effects but currently, there is insufficient information to do so”.*

8. **In general: how many observational studies recorded actual applications in relation to the observations?**

To develop this point, we added the following paragraph in the section “Assessment of residential exposures in observational measurement studies” (lines 242-243 of the original manuscript):

*“Four small-scale observational studies measured pesticide exposure during actual applications. These studies were conducted in association with local farmers to define the nature of the substances applied and the spraying locations. Five large-scale studies conducted in California obtained this information from the PUR dataset. Other observational studies did not report actual pesticide applications, but a majority measured pesticide exposure during a spraying season.”*

9. **Under Strenght and weaknesses: Check the grey literature on the Dutch resident study: https://www.bestrijdingsmiddelen-omwonenden.nl/documenten/onderzoeksrapport-obo-1.**

The research on residents' exposure to pesticides in the Netherlands was identified in this scoping review through a search of the grey literature. This work was considered as an observational measurement study. The reference of this report is available in the complete list of the selected studies in Supplemental file S1.

**C/ Additional corrections (not required by the reviewers)**

Changes have been made in the affiliations of the authors.

Lines 254 of the initial manuscript 🡺 we changed the title of the Table “*Table 3. Assessment of exposure in observational ~~exposure~~ measurement studies*”

Lines 242 to 243 of the initial manuscript 🡺 the definition of buffer has been moved to the previous section to clarify the article (lines 213-215 in the revised manuscript)

Lines 352 of the initial manuscript 🡺 we detected an error and made a change of reference in the section “Discussion”.

Lines 491 to 684 of the initial manuscript 🡺 references have been actualized.
